# Cognitive Function in Ambulatory Patients with Systolic Heart Failure: Insights from the Warfarin versus Aspirin in Reduced Cardiac Ejection Fraction (WARCEF) Trial

**DOI:** 10.1371/journal.pone.0113447

**Published:** 2014-11-26

**Authors:** Susan Graham, Siqin Ye, Min Qian, Alexandra R. Sanford, Marco R. Di Tullio, Ralph L. Sacco, Douglas L. Mann, Bruce Levin, Patrick M. Pullicino, Ronald S. Freudenberger, John R. Teerlink, J. P. Mohr, Arthur J. Labovitz, Gregory Y. H. Lip, Conrado J. Estol, Dirk J. Lok, Piotr Ponikowski, Stefan D. Anker, John L. P. Thompson, Shunichi Homma, for the WARCEF Investigators

**Affiliations:** 1 Division of Cardiology, Department of Medicine, State University of New York Upstate Medical University, Buffalo, New York, United States of America; 2 Division of Cardiology, Department of Medicine, Columbia University Medical Center, New York, New York, United States of America; 3 Department of Biostatistics, Columbia University Mailman School of Public Health, New York, New York, United States of America; 4 Department of Neurology, University of Miami Miller School of Medicine, Miami, Florida, United States of America; 5 Department of Medicine, Washington University, St. Louis, Missouri, United States of America; 6 Kent Institute of Medicine and Health Sciences, University of Kent, Canterbury, United Kingdom; 7 Division of Cardiology, Department of Medicine, Lehigh Valley Hospital, Allentown, Pennsylvania, United States of America; 8 Section of Cardiology, Department of Medicine, San Francisco Veterans Affairs Medical Center, University of California San Francisco, San Francisco, California, United States of America; 9 Department of Neurology, Columbia University Medical Center, New York, New York, United States of America; 10 Department of Cardiovascular Medicine, University of South Florida, Tampa, Florida, United States of America; 11 University of Birmingham Centre for Cardiovascular Sciences, City Hospital, Birmingham, United Kingdom; 12 Centro Neurológico de Tratamiento y Rehabilitación, Buenos Aires, Argentina; 13 Department of Cardiology, Deventer Hospital, Deventer, Overijssel, The Netherlands; 14 Department of Heart Diseases, Wroclaw Medical University, Military Hospital, Wroclaw, Lower Silesia, Poland; 15 Department of Innovative Clinical Trials, University Medical Centre Göttingen, Göttingen, Lower Saxony, Germany; Kurume University School of Medicine, Japan

## Abstract

We sought to determine whether cognitive function in stable outpatients with heart failure (HF) is affected by HF severity. A retrospective, cross-sectional analysis was performed using data from 2, 043 outpatients with systolic HF and without prior stroke enrolled in the Warfarin versus Aspirin in Reduced Cardiac Ejection Fraction (WARCEF) Trial. Multivariable regression analysis was used to assess the relationship between cognitive function measured using the Mini-Mental Status Exam (MMSE) and markers of HF severity (left ventricular ejection fraction [LVEF], New York Heart Association [NYHA] functional class, and 6-minute walk distance). The mean (SD) for the MMSE was 28.6 (2.0), with 64 (3.1%) of the 2,043 patients meeting the cut-off of MMSE <24 that indicates need for further evaluation of cognitive impairment. After adjustment for demographic and clinical covariates, 6-minute walk distance (*β*-coefficient 0.002, p<0.0001), but not LVEF or NYHA functional class, was independently associated with the MMSE as a continuous measure. Age, education, smoking status, body mass index, and hemoglobin level were also independently associated with the MMSE. In conclusion, six-minute walk distance, but not LVEF or NYHA functional class, was an important predictor of cognitive function in ambulatory patients with systolic heart failure.

## Introduction

Reduced cognitive function is common in patients with heart failure,[1]–[9] and the ensuing impairment of executive function, memory and attention can adversely affect patients’ quality of life and capacity for self-care. [8], [10] In addition to comorbidities such as hypertension and diabetes and psychosocial factors such as depression, [11] decreased cerebral perfusion due to cardiac dysfunction has been proposed as a key mechanism for the association between heart failure and cognitive impairment.[2], [3], [10]–[12] Supporting this hypothesis, imaging studies have demonstrated organic changes in brain areas responsible for cognitive and executive functions in patients with heart failure, [13] and have shown that cardiac index is negatively associated with markers of brain aging in healthy individuals. [12] Furthermore, it has been suggested that heart failure severity can be an important predictor of cognitive function. [2], [5], [6], [10] However, many of these studies have important limitations, including small sample sizes [5] and potential confounding due to the restriction of enrollment to hospitalized or elderly individuals. [1], [2], [4], [6], [7] A better understanding of how heart failure status affects cognitive function is thus needed, and can potentially provide insights to improve chronic management of heart failure.

The Warfarin versus Aspirin in Reduced Cardiac Ejection Fraction (WARCEF) trial, [14] which followed a broad range of individuals with medically managed chronic systolic heart failure who were in sinus rhythm, provides a unique opportunity to address this gap in knowledge. We undertook the present analysis of the WARCEF trial to characterize the predictors of cognitive status as measured by the Mini-Mental State Examination (MMSE), and to determine whether there is an independent association between cognitive function and measures of heart failure severity, as measured by left ventricular ejection fraction (LVEF), New York Heart Association (NYHA) functional class, and 6-minute walk distance.

## Methods

The protocol for the randomized, double blinded WARCEF trial (http://www.ClinicalTrials.gov No. NCT00041938) has been described previously. [14], [15] Briefly, patients with left ventricular ejection fraction (LVEF) ≤35% and who were in sinus rhythm at time of enrollment were randomized to receive warfarin (target INR 2.75, with acceptable target range of 2.0 to 3.5) or aspirin (325 mg daily). Additional eligibility criteria included being 18 years or older, having no contraindications to warfarin therapy, having a modified Rankin score of 4 or less (on a scale of 0 to 6, with higher scores indicating more severe disability), and treatment with a beta blocker, an angiotensin-converting-enzyme (ACE) inhibitor or angiotensin-receptor blocker (ARB), or hydralazine and nitrates. Patients were excluded if they had a clear indication for warfarin or aspirin, or if they had a condition that conferred a high risk of cardiac embolism, such as atrial fibrillation, a mechanical cardiac valve, endocarditis, or an intracardiac mobile or pedunculated thrombus. Patients were also excluded if they were unable to follow an outpatient study protocol, or if they were unable to provide informed consent. Patients in any NYHA functional class were eligible, although patients in NYHA class I could account for no more than 20% of the total sample. A total of 2,305 participants were recruited from 168 centers in 11 countries from October 2002 to January 2010. The investigation conforms with the principles outlined in the Declaration of Helsinki, [16] and the study was approved by Institutional Review Boards at the coordinating centers for all sites. All subjects provided informed consent. For this analysis, we further excluded 248 participants with prior ischemic stroke, and 14 others who did not have complete MMSE assessment. The final sample for analysis thus included 2,043 participants.

### Cognitive Function, Heart Failure Severity, and Other Covariates

All data used for this analysis were collected at time of enrollment for the WARCEF trial. The primary outcome of this post hoc analysis was cognitive function using the MMSE at the time of enrollment. The MMSE is a brief, validated, 30-point questionnaire commonly used to screen for cognitive impairment. [17] In the WARCEF trial, the MMSE was administered in a standardized fashion in the native language of each participant by trained staff at each individual site. Importantly, results of the MMSE were not directly used to determine patient eligibility for the WARCEF trial.

For this analysis, measures of heart failure severity included LVEF (measured by quantitative echocardiography or radionuclide or contrast ventriculography), 6-minute walk distance, [18], [19] and NYHA functional class. LVEF measurements were obtained by central reading of tests performed at participating study sites by experienced observers blinded to randomization status of trial participants. For each participant, 6-minute walk distance was obtained by study coordinators who measured distance walked over a 6-minute period following a standardized protocol, [18] and NYHA functional class was determined on the basis of self-reported symptoms by investigators at each study site.

Additional baseline demographic covariates that were collected include age, sex, and education level (dichotomized for the current analysis as less than high school or high school graduate and above). Health behaviors were assessed by self-report at time of enrollment, including smoking status (never, former, or current smokers) and alcohol consumption (never, former consumption of >2 oz/day, or current consumption of >2 oz/day). Clinical covariates were determined at the initial study visit, including medical comorbidities (history of atrial fibrillation, hypertension, diabetes mellitus, ischemic cardiomyopathy), medications (beta-blockers and ACE-inhibitors/ARBs), body mass index (weight in kilograms divided by height in meters squared), baseline systolic blood pressure, and serum creatinine and hemoglobin levels (measured within 30 days of study enrollment).

### Statistical Analysis

Descriptive statistics were provided for baseline MMSE, heart failure severity measures, and demographic and clinical covariates described above. We further calculated the proportion of patients with a MMSE of <27, the cut-off for abnormal cognitive function, and the proportion of patients with a MMSE of <24, a cut-off commonly used to indicate need for further evaluation of significant cognitive impairment. [20] For subsequent regression analyses, the MMSE was treated as a continuous variable as it was clustered around higher scores in the WARCEF population. Univariable regression analysis was performed with the MMSE as the dependent variable and individual predictors, including heart failure severity measures and other demographic and clinical covariates, as independent variables. Subsequently, all predictors were entered into a multiple regression model with the MMSE as the dependent variable. For all regression analyses, NYHA functional classes III and IV were combined due to the small number of participants with NYHA functional class IV. For continuous covariates, the regression *β*-coefficient represents the expected point-increase in mean MMSE for every one-unit increase of the covariate. For categorical covariates, the regression *β*-coefficient represents the difference in mean MMSE between the current category and the reference category. There was 9% (180/2043) missing data on 6-minute walk distance, 8% (166/2043) on hemoglobin, and 3% (58/2043) on hypertension; less than 10 values were missing on all other variables. Therefore, multiple imputation was used to account for missing covariate data. Specifically, we created five data sets using a sequential regression imputation method, [21] performed regression analyses on each data set, and subsequently combined the results to produce the reported regression *β*-coefficients and *t*-test based 95% confidence intervals and p-values using the method described by Rubin. [22] All statistical analyses were performed using SAS software (version 9.2; SAS Institute, Cary, NC).

## Results

Among the 2,043 WARCEF patients included in this analysis, the mean (SD) age was 60.8 (11.3) years, and 1634 (80%) were male. Other demographic and clinical covariates are described in **Table 1**. Of note, a high proportion of participants were treated with evidence-based heart failure therapy, including 90.3% who were on beta-blockers and 98.5% who were on ACE-inhibitors or ARBs.

**Table 1:** Baseline characteristics of participants in the WARCEF trial included for analysis.

	All patients (N = 2043)
Cognitive function	
MMSE, score	28.6 (2.0)
Cognitive impairment (MMSE<24)	64/2043 (3.1%)
Cognitive impairment, adjusted for education*	29/2043 (1.4%)
Heart Failure Severity Measures	
Ejection fraction, %	24.6 (7.6)
6 minute walk, m	355.9 (146.7)
NYHA classification	
I	271/2034 (13.3%)
II	1145/2034 (56.3%)
III	594/2034 (29.2%)
IV	24/2034 (1.2%)
Demographic covariates	
Age, years	60.8 (11.3)
Male gender	1634/2043 (80.0%)
High school graduate or greater	1147/2039 (56.3%)
Smoking status	
Current smoker	352/2042 (17.2%)
Former smoker	1054/2042 (51.6%)
Alcohol consumption	
Current consumption,>2 oz per day	517/2042 (25.3%)
Previous consumption,>2 oz per day	433/2042 (21.2%)
Medical comorbidities	
History of atrial fibrillation	77/2041 (3.8%)
Hypertension	1186/1985 (59.8%)
Diabetes Mellitus	616/2040 (30.2%)
Ischemic cardiomyopathy	878/2039 (43.1%)
Body mass index, kg/m^2^	29.2 (6.0)
Systolic blood pressure, mmHg	123.5 (18.6)
Serum creatinine, mg/dL	1.2 (0.3)
Hemoglobin, g/dL	14.1 (1.5)
Heart Failure Medication	
Beta blocker	1843/2042 (90.3%)
ACE-inhibitor or ARB	2011/2041 (98.5%)

Values are expressed as mean (SD) or number (%), where appropriate.

*Cognitive impairment adjusted for education is defined as a MMSE score of <20 for patients with less than high school education, or <24 for high school graduates or above.

Abbreviations: MMSE, Mini-Mental State Examination; NYHA, New York Heart Association Class; ACE, angiotensin converting enzyme; ARB, angiotensin receptor blocker.

### Cognitive Function and Heart Failure Severity

**Figure 1:**
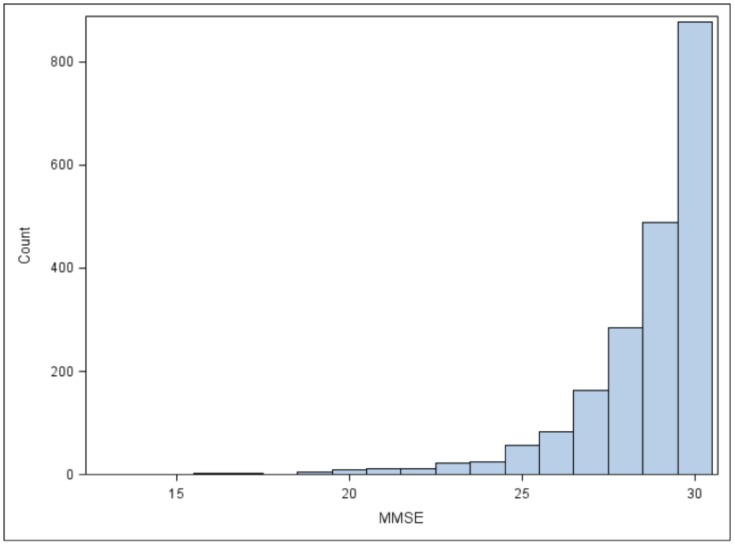
Distribution of the Mini-Mental Status Exam scores in WARCEF patients included for analysis.

The distribution of the MMSE scores was skewed towards higher values, as shown in **Figure 1**. 228 (11.2%) of 2043 patients had MMSE score of <27, suggestive of abnormal cognitive function, while 64 (3.1%) patients had MMSE score of <24, indicating need for further evaluation of significant cognitive impairment. [20] For heart failure severity, the mean (SD) for LVEF was 24.6% (7.6%), and the mean (SD) distance for the 6-minute walk test was 356 meters (150 meters). Slightly more than two thirds of participants were in NYHA class I or II, with most of the remainder having NYHA class III symptoms. (**Table 1**).

### Univariable and Multivariable Determinants of Cognitive Function

In univariable regression analysis, the MMSE score was significantly associated with both 6-minute walk distance (*β*-coefficient 0.002, p<0.0001) and having NYHA class III or IV symptoms (*β*-coefficient −0.460, p = 0.001), but not LVEF (*β*-coefficient 0.006, p = 0.264) (**Table 2**). Other predictors that were significant in univariable models included age, high school education or above, current or former smoking, current alcohol consumption of >2 oz per day, hypertension, diabetes mellitus, body mass index, and serum creatinine and hemoglobin levels. In the full multiple regression model, however, the only marker of heart failure severity that independently predicted the MMSE score was increased 6-minute walk distance (*β*-coefficient 0.002, p<0.0001). Increasing age remained negatively associated with the MMSE after adjusting for other variables, while high school education or above, higher body mass index, current or former smoking status, and higher serum hemoglobin were positively associated with the MMSE score in the fully adjusted model.

**Table 2:** Predictors of cognitive function (as measured by the Mini-Mental Status Exam) in univariable and multivariable regression models.

	Univariable model (unadjusted)		Multivariable model	
	*β*-coefficient (95% CI)	p-value	*β*-coefficient (95% CI)	p-value
Heart Failure Severity Measures				
Ejection fraction, %	0.006 (−0.005, 0.018)	0.264	0.007 (−0.004, 0.018)	0.207
6 minute walk, m	**0.002 (0.002, 0.003)**	**<0.0001**	**0.002 (0.001, 0.002)**	<**0.0001**
NYHA classification				
I	Ref		Ref	
II	−0.128 (−0.389, 0.133)	0.336	−0.031 (−0.286, 0.225)	0.813
III or IV	−**0.460 (**−**0.741, −0.179)**	**0.001**	−0.167 (−0.451, 0.117)	0.250
Demographic covariates				
Age, years	−**0.028 (**−**0.036, −0.021)**	**<0.0001**	−**0.019 (**−**0.027, −0.011)**	<**0.0001**
Male gender	0.132 (−0.082, 0.345)	0.227	−0.136 (−0.365, 0.092)	0.242
High school graduate or greater	**0.594 (0.424, 0.764)**	**<0.0001**	**0.511 (0.342, 0.680)**	<**0.0001**
Smoking status				
Never smoker	Ref		Ref	
Current smoker	**0.444 (0.188, 0.699)**	**0.001**	**0.321 (0.061, 0.582)**	**0.016**
Former smoker	**0.201 (0.007, 0.394)**	**0.042**	**0.204 (0.009, 0.399)**	**0.041**
Alcohol consumption				
None	Ref		Ref	
Current consumption,>2 oz per day	**0.306 (0.100, 0.511)**	**0.004**	0.181 (−0.025, 0.386)	0.085
Previous consumption,>2 oz per day	−0.035 (−0.253, 0.184)	0.756	−0.133 (−0.355, 0.088)	0.239
Medical comorbidities				
History of atrial fibrillation	−0.186 (−0.634, 0.263)	0.417	−0.056 (−0.490, 0.378)	0.801
Hypertension	−**0.209 (**−**0.384, −0.035)**	**0.019**	−0.124 (−0.306, 0.058)	0.182
Diabetes Mellitus	−**0.265 (**−**0.451, −0.080)**	**0.005**	−0.147 (−0.336, 0.043)	0.130
Ischemic cardiomyopathy	−0.123 (−0.295, −0.050)	0.163	0.041 (−0.133, 0.214)	0.647
Body mass index, kg/m^2^	**0.021 (0.007, 0.035)**	**0.003**	**0.015 (0.000, 0.031)**	**0.047**
Systolic blood pressure, mmHg	0.0003 (−0.004, 0.004)	0.897	0.002 (−0.003, 0.007)	0.452
Serum creatinine, mg/dL	−**0.290 (**−**0.552, −0.029)**	**0.029**	0.026 (−0.246, 0.298)	0.853
Hemoglobin, g/dL	**0.160 (0.104, 0.215)**	**<0.0001**	**0.085 (0.026, 0.145)**	**0.005**

For the multivariable model, the adjusted R^2^ was 0.073.

## Discussion

In this retrospective, cross-sectional analysis of WARCEF participants with stable, medically managed systolic heart failure who were in sinus rhythm and who did not have prior ischemic strokes, we found that the only measure of heart failure severity that was independently associated with cognitive function was the 6-minute walk distance: LVEF and NYHA functional class were not significant predictors of the MMSE score after adjustment for potential demographic and clinical confounders. Furthermore, younger age and higher educational status were both independently associated with higher MMSE scores, as was current or former smoking, body mass index, and serum hemoglobin level.

Our study extends the literature on the relationship between heart failure status and cognitive function. Previous studies have shown inconsistent associations between cognitive function and measures of heart failure severity such as LVEF and NYHA function class. [5], [6], [23] Although one study by Baldasseroni and colleagues demonstrated a positive association between 6-minute walk distance and cognitive function, their analysis was limited by a small sample size of only 80 elderly patients with heart failure. [24] In contrast, our analysis confirmed this relationship in a large sample of well-characterized, stable ambulatory heart failure patients. Furthermore, our results are also consistent with previous observations that cardiovascular fitness and exercise capacity are important determinants of cognitive function in both patients with heart failure [25] and the general population. [26] Taken together, our findings suggest that the 6-minute walk distance, which has been shown to be a powerful predictor of prognosis in patients with heart failure, [18], [27] is an objective indicator of heart failure status that was also strongly associated with cognitive function in this patient population.

Reduced cardiac output leading to decreased cerebral perfusion is thought to contribute to decreased cognitive function in patients with heart failure, [3], [12] but it is likely that other mechanisms also play a role in the relationship between 6-minute walk distance and cognitive function that we observed in WARCEF participants. The 6-minute walk distance integrates clinical and physiological aspects of general fitness, and comorbidities such as hypertension, diabetes, and atherosclerotic vascular disease could reduce both exercise capacity and cognitive function in patients with heart failure. [11] Nonetheless, although comorbidities such as hypertension and diabetes were associated with the MMSE for WARCEF patients in univariable analyses, these associations were no longer significant in the multivariable model, while the association between 6-minute walk distance and the MMSE remained. Given the limitations of the retrospective, cross-sectional design of the current study, future research will need to confirm our findings and more precisely elucidate the mechanisms by which heart failure influences cognitive function.

In addition to the expected effect of age and education, we also found that hemoglobin level, body mass index, and current or former smoking status were independent predictors of the MMSE in this patient population. Anemia has previously been shown to be associated with cognitive dysfunction in elderly individuals. [28] Our results suggest that this relationship may also be true in patients with heart failure, potentially representing another mechanism for the increased risk of mortality and heart failure hospitalization conveyed by anemia that have been well-described in this patient population. [29] Similarly, low body mass index has been shown to correlate with cognitive decline, [30] and may reflect cardiac cachexia and increased vulnerability to cognitive dysfunction in patients with heart failure. It is however surprising that both current and former smokers had higher MMSE scores compared to never-smokers in our sample, a finding that contrasts with those from prior epidemiological studies. [31] We speculate that this may reflect confounding of the relationship between smoking status and the MMSE because of other factors, as current smokers were younger and had a lower prevalence of diabetes than non-smokers in our sample, and both current and former smokers had higher hemoglobin levels and lower systolic blood pressure than non-smokers (data not shown).

It is worth noting that in our sample of stable outpatients with heart failure, only a small proportion of patients had MMSE of <27 (11.2%) or <24 (3.1%). These findings are in contrast to prior studies suggesting that cognitive dysfunction could affect more than half of patients with heart failure. [1], [2], [4], [6], [9] A likely reason for this discrepancy is that, as in other heart failure clinical trials, most patients enrolled in WARCEF were younger than heart failure patients followed in the community. [32] Furthermore, the exclusion from our sample of patients who could not provide informed consent likely reduced the proportion of patients with significant cognitive impairment. The MMSE may also be relatively insensitive for detecting cognitive impairment in patients with heart failure, who may often have subtle deficits in cognitive domains such as recall and executive function. [9] Despite these limitations, however, our analysis was able to identify that the 6-minute walk distance is an independent predictor of higher MMSE in ambulatory heart failure patients.

There are several other limitations to our study. As a retrospective, secondary analysis of the WARCEF trial, our results are necessarily hypothesis generating. Since approximately 80% of WARCEF participants were male, our findings may also not be generalizable to female patients with heart failure. Furthermore, we analyzed data collected during the conduct of the WARCEF trial, and could not account for psychosocial factors such as depression that could influence assessment of cognitive function. Similarly, the WARCEF trial protocol did not include formal neuropsychiatric testing or invasive hemodynamic assessment, which are more definitive measurements of cognitive function and cardiac output, respectively. We assessed cognitive function at enrollment only, and the cross-sectional design of this analysis does not allow us to determine whether improvement or deterioration of cognitive function may correlate with changes in heart failure status. [10] Finally, given the WARCEF trial only included heart failure patients in sinus rhythm, our findings cannot be extrapolated to individuals with heart failure and other arrhythmias.

## Conclusion

In this retrospective, cross-sectional analysis of stable, medically managed outpatients with systolic heart failure, we found that the 6-minute walk distance, a measure of heart failure severity, was an important predictor of cognitive function as measured by the MMSE. Our findings may be useful for future efforts to understand and treat cognitive impairment in this patient population.
